# Current Evidence and Future Perspectives to Implement Continuous and End-Ischemic Use of Normothermic and Oxygenated Hypothermic Machine Perfusion in Clinical Practice

**DOI:** 10.3390/jcm12093207

**Published:** 2023-04-29

**Authors:** Maxime Foguenne, Serena MacMillan, Philipp Kron, Jay Nath, Arnaud Devresse, Martine De Meyer, Mourad Michel, Sarah Hosgood, Tom Darius

**Affiliations:** 1 Surgery and Abdominal Transplant Unit, Department of Surgery, University Clinics Saint Luc, Université Catholique de Louvain, 1200 Brussels, Belgium; 2 Department of Surgery, University of Cambridge, Addenbrooke’s Hospital, Cambridge CB2 0QQ, UK; 3 Department of Surgery and Transplantation, Swiss HPB Center, University Hospital Zurich, 8091 Zurich, Switzerland; 4 Department of Renal Transplantation, Southmead Hospital Bristol, Bristol BS10 5NB, UK; 5 Department of Nephrology, University Clinics Saint-Luc, Université Catholique de Louvain, 1200 Brussels, Belgium

**Keywords:** normothermic machine perfusion, hypothermic machine perfusion, kidney preservation, oxygenation, mitochondria, organ preservation, kidney assessment, kidney reconditioning, kidney modulation

## Abstract

The use of high-risk renal grafts for transplantation requires the optimization of pretransplant assessment and preservation reconditioning strategies to decrease the organ discard rate and to improve short- and long-term clinical outcomes. Active oxygenation is increasingly recognized to play a central role in dynamic preservation strategies, independent of preservation temperature, to recondition mitochondria and to restore the cellular energy profile. The oxygen-related decrease in mitochondrial succinate accumulation ameliorates the harmful effects of ischemia-reperfusion injury. The differences between normothermic and hypothermic machine perfusion with regard to organ assessment, preservation, and reconditioning, as well as the logistic and economic implications, are factors to take into consideration for implementation at a local level. Therefore, these different techniques should be considered complementary to the perfusion strategy selected depending on functional intention and resource availability. This review provides an overview of the current clinical evidence of normothermic and oxygenated hypothermic machine perfusion, either as a continuous or end-ischemic preservation strategy, and future perspectives.

## 1. Introduction

One of the current major challenges in kidney transplantation (KT) is the deteriorating quality of available deceased organs and the constantly growing gap between available donor grafts and patients on the waiting list. This results in the death of patients while they are awaiting a transplant [1]. Therefore, high-risk organs are nowadays commonly accepted for KT and defined as grafts originating from donation after circulatory death (DCD) or from brain death donors (DBDs) with expanded criteria donors (ECDs). By definition, these kidneys are more suspectible to ischemia-reperfusion injury (IRI), which is a well-known risk factor for primary nonfunction (PNF), delayed graft function (DGF), and graft failure [2,3,4,5]. A significant number of kidneys are discarded in the pretransplant period due to the lack of objective criteria to assess organ quality and the perceived limitations of organ resuscitation and repair during preservation [6,7,8,9,10,11]. Therefore, from the beginning of the twenty-first century, a revival of machine perfusion strategies has been observed and is still ongoing. Indeed, it permits us to assess the viability of the kidney and to improve organ preservation, resulting in an increased organ utilization rate. Machine perfusion strategies can be applied before organ procurement. As blood viscosity and rheology change below 20 °C, we differentiate hypothermic regional perfusion (≤20 °C) and normothermic regional perfusion (NRP, >20 °C). Clinical experience with hypothermic regional perfusion is rare and dates before 2005. In contrast, the use of NRP for abdominal but also thoracic organs in DCD is increasing worldwide and is a safe alternative to in situ cooling and rapid procurement. In addition, NRP reduces post-transplant complications, in particular, biliary complications in controlled DCD, compared to in situ cold preservation. No data after NRP for kidney and pancreas exists at this moment compared to in situ cold preservation, but there is no evidence that NRP is detrimental [12,13]. In contrast, machine perfusion strategies can also be applied after organ procurement, either in a continuous way (from the moment of kidney procurement until the moment of transplantation) or as an end-ischemic preservation strategy (e.g., after a preceding period of static cold storage preservation). The two most widely implemented ex vivo preservation strategies are hypothermic and normothermic machine perfusion (HMP and NMP).

Ex vivo kidney evaluation (quality assessment) during NMP is feasible thanks to the near-physiological created environment. In addition, there is the opportunity for the delivery of novel therapeutics to enhance organ preservation and reconditioning, thus decreasing the harmful effects of IRI after the anoxic hypothermic conditions of static cold storage (SCS) or traditional HMP [14,15,16]. NMP requires either blood-based oxygenation or an O_2_ carrier. Furthermore, the acid–base balance, nutrient supply, and disposition of products of metabolism must be closely monitored. Moreover, in case of pump failure, the risk of organ loss is high. In contrast, HMP is less complex and cheaper, and the risk of organ loss due to pump failure is minimal. Therefore, HMP is currently the most established perfusion technique for deceased donor kidneys. This review provides an overview of the current clinical evidence of ex vivo oxygenated HMP and NMP approaches, either as continuous or end-ischemic preservation strategies and their future perspectives.

## 2. Mitochondria Play a Central Role in Dynamic Preservation Strategies

**Figure 1:**
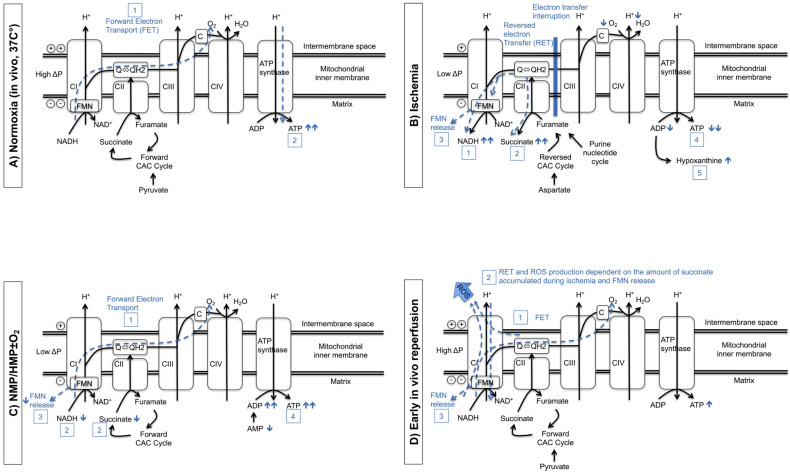
Mitochondrial changes during oxygenated NMP and HMP through the process of kidney preservation and transplantation. (**A**), In a normoxic state, namely, before starting the procurement procedure, forward electron transport (FET) (dotted line) (1) drives the proton pumps (CI, CIII, CIV) against a charge gradient at the oxidative phosphorylation chain. ATP is generated by ATP synthase, driven by proton back-flow (dotted line) (2) [24,31]. (**B**), During ischemia, electron transfer is interrupted, but CI continues to pump protons. This results in the accumulation of electron carriers (firstly NADH (1), followed secondly by succinate (2) (an acid citric metabolite). In addition, the over-reduction of flavin via reversed electron transport (RET) at CI results in FMN release (3) [30,32]. This leads to a decrease in ATP and ADP (4) and an increase in purine metabolites (5) [24,31]. (**C**), The level of perfusate oxygen concentration determine the degree of FET during NMP and HMP ± O_2_ (1). This results in succinate consumption and NADH oxidation (2), reduced FMN release (3), and restoration of ADP/ATP stock (4) [26,31]. (**D**), ATP is generated by FET (1) during early in vivo reperfusion. Succinate is rapidly consumed, which results in excessive quantities of ubiquinol (QH2), impairing further FET. This impairment, combined with ischia-related acidosis, results in a RET at CI (2) [24,34]. During this reaction, ROS are generated from reduced or semi-reduced FMN located at the nucleotide-binding site. ROS are also produced by a non-enzymatic reaction between reduced FMN and molecular oxygen [30]. Figure and figure legend are slightly adapted but based on Figure 3 of T. Darius et al. [35].

During SCS and traditional HMP, metabolic processes are suppressed to reduce cellular oxygen demand and adenosine triphosphate (ATP) depletion [16]. Under hypothermic conditions (4 °C), oxygen consumption is reduced by 90–95% compared to that at normal body temperature [17,18,19]. However, during cold preservation, a decrease of 90% in oxygen consumption was observed in perfusion fluid oxygen levels after 2 h [20]. Prolonged hypoxia and subsequent IRI have been recognized as a cause of cellular dysfunction and DGF after KT [21,22]. During ischemia, succinate originating from the citric acid cycle (CAC) accumulates in the mitochondria. During subsequent in vivo reperfusion under normothermic conditions, the succinate accumulation is a main contributor to superoxide generation by reversed electron transport (RET) [23,24,25]. This mechanism was described by Dutkowski et al. during the assessment of different human liver preservation strategies [26], and a similar mechanism was confirmed during kidney perfusion in a porcine autotransplant model [27]. To correct pre-existing oxygen debt (acquired by donor and procurement-related factors) and given the oxygen consumption by the ex vivo perfused kidney, independent of preservation temperature, additional oxygenation during machine perfusion is now considered to play a central role in dynamic preservation strategies to recondition mitochondria and to restore the cellular energy profile. Restoring the mitochondrial energy profile (correction of ATP and decrease the ischemic accumulation of mitochondrial succinate) intends to improve kidney preservation and decrease the degree of IRI. Active oxygenation during NMP and HMP has an impact on mitochondrial metabolism, as illustrated in Figure 1. In vivo, cellular ATP is mainly produced from mitochondrial oxidative phosphorylation and is the final electron acceptor in oxygenated conditions (Figure 1A) [18,24,28,29,30,31]. Complex I (CI), containing 45 subunits, is composed of 8 different iron-sulfur clusters and bounded tightly but non-covalently to flavin mononucleotide (FMN) [30]. Electrons of reduced nicotinamide adenine dinucleotide (NADH) are transferred to flavin, while the pathway for the forward electron transport (FET) to the bulk ubiquinone (Q) is provided by the iron-sulfur clusters [30]. Hypoxia causes an interruption of FET, replaced by RET, which results in the accumulation of succinate, purine metabolites, and NADH, combined with FMNH_2_ release and a drop of ATP/ADP reserve) (Figure 1B) [24,26,30,31,32]. Historically, HMP was conceptualized without active oxygenation, explaining the absence of any oxygen carrier in preservation solutions. Therefore, during HMP, oxygen reaches the tissue by diffusion. In contrast, red blood cells usually serve as oxygen carriers in NMP. In both MP strategies, oxygen promotes physiological mitochondrial processes with evidence of FET activity (Figure 1C) [26,27,31,33]. In the presence of oxygen, FET generates ATP during early in vivo reperfusion. Accumulated succinate is consumed, generating excessive ubiquinol (QH2), impairing further FET. The degree of ischemic-accumulated succinate levels determines the production of reactive oxygen species (ROS) as a result of RET at complex I (Figure 1D) [24,34].

## 3. Hypothermic Machine Perfusion

### 3.1. Establishment of Hypothermic Machine Perfusion on the Kidney

Early research on HMP emerged half a century ago [36,37]. The first clinical machine perfusion device was created by Folkert O Belzer in 1968 and used during the first successful human transplantation of a 17 h hypothermically machine-perfused kidney [38]. During HMP, the continuous perfusion of the kidney graft with a cold preservation solution (1–10 °C) results in a continuous flush of the microcirculation and prevents the accumulation of toxic metabolites. Worldwide, University of Wisconsin solution modified for machine perfusion is the most used solution. This is an acellular hypertonic fluid not containing an oxygen carrier. Regarding HMP perfusion devices, numerous portable devices are commercially available, including the Kidney Assist Transporter (Organ Assist BV, Groningen, The Netherlands), the WAVES machine (Institut Georges Lopez, Lissieu, France), and the LifePort Kidney Transporter (Organ Recovery Systems, Chicago, IL, USA). Otherwise, some companies developed non-portable ones such as the RM3 device (Waters Medical Systems, Rochester, MN, USA) and the VitaSmart (Bridge to Life, Northbrook, IL, USA). Some devices are flow-driven (e.g., RM3 system pressure-driven), while others are pressure-driven (e.g., LifePort and Kidney Assist). The debate is still open about the eventual superiority of one machine over the other. Nevertheless, some evidence illustrates a minor advantage of pressure-driven above flow-driven devices because of the lower pressures and thereby decreasing pressure-related injury during perfusion [39,40]. A pulsatile renal artery pressure of 25–30 mmHg appears to be ideal for kidney perfusion [41,42,43]. Currently, some HMP devices incorporated active oxygenation as standard, while others can easily be modified to include an oxygenator.

The beneficial effects of HMP are numerous. Namely, it is responsible for flow-related mechanical vasodilatation and molecular vasoprotection. Indeed, protective endothelial genes, hypoxia-inducible factor-α, and nitric oxide signaling are overexpressed. In contrast, fibrosis and innate immunity are decreased. Similarly, pro-inplammatory cytokines, vementin, endothelin, Toll-like receptor 4, and High-Mobility Group Box 1 (HMGB1) gene expression are underexpressed [44,45,46,47,48,49,50,51,52].

### 3.2. HMP as an Alternative to Static Cold Storage

A recent meta-analysis demonstrated the superiority of HMP kidneys compared to SCS, showing reduced rates of PNF and DGF and an improved one-year graft survival (OR:1.61 95% CI: 1.02 to 2.53, *p* = 0.04) [53]. This was concordant with another meta-analysis in 2019 that demonstrated the superiority of HMP over SCS for both DBD and DCD kidneys [54]. The incidence of DGF is higher for grafts from DCD donors as compared to DBD, and therefore, fewer perfusions are needed to prevent one DGF episode (7.26 for DCD versus 13.60 for DBD grafts) [54]. Based on both meta-analyses, continuous HMP is superior to SCS. Even when applied for short cold ischemia times (less than 10 h), HMP demonstrated proven superiority above SCS alone [55]. However, HMP should be applied for the entire cold ischemic period, as end-ischemic application after a preceding period of SCS has not demonstrated any clinical benefit [43,56]. In addition, graft failure and DGF risks were at lower costs reduced by HMP as compared to SCS [57].

### 3.3. Kidney O_2_ Delivery during HMP?

Preclinical evidence outlines the key role of oxygen during HMP [46,58,59,60,61,62]. However, the optimal route of applying oxygen is still under debate. Currently, two oxygenation techniques exist for clinical application: membrane oxygenation and bubble and surface oxygenation [35].

#### 3.3.1. Membrane Oxygenation

Because of their application in other clinical devices (e.g., extracorporeal oxygenator in cardiac surgery), hollow fiber membrane oxygenators were incorporated in commercial devices such as the Kidney Assist Transporter (Organs Assist, Groningen, The Netherlands) and the VitaSmart system (Bridge to Life, London, UK), as well as in non-commercial non-transportable models, as recently described in an Italian trial [63]. These devices allow us to achieve a partial oxygen pressure (pO_2_) of at least 80–100 kPa (600–750 mmHg) [64,65,66]. Membrane oxygenation principles rely on O_2_ diffusion through a thin gas-permeable membrane from the gas compartment into the perfusion fluid. The oxygenated perfusion solution is administrated by arterial cannulation, and oxygen reaches the renal cellular compartment by diffusion [35].

#### 3.3.2. Bubble and Surface Oxygenation

A more simple and cheaper alternative to membrane oxygenation is bubble and surface oxygenation. Its oxygen transfer capacity is proven to be more than sufficient to sustain the aerobic metabolism of a single kidney at ±4 °C [35,67,68]. This technique has been previously described in detail [35] and is commercially available on the LifePort Kidney Transporter^®^ (Organ Recovery Systems, Chicago, IL, USA). Since the first clinical application in humans in 2022, other European centers have introduced this oxygenation technique in their clinical HMP programs [69]. The clinical implementation of bubble and surface oxygenation to raise the dissolved perfusate O_2_ concentration is based on four principles [35]. First, the solubility of oxygen is inversely proportional to the temperature [29,70]. Secondly, bubble oxygenation increases proportionally with oxygen volume and inversely proportionally with bubble size. This results in highly effective O_2_ transfer (achieving perfusate pO_2_ of at least 80–100 kPa (600–750 mmHg) after 15 min of bubble oxygenation comparable to membrane oxygenation) [33]. Thirdly, O_2_ diffuses slowly across the perfusate surface from the gaseous compartment into the perfusion fluid according to Henry’s law. The quantity of O_2_ diffusing into the perfusate will be proportional to the gaseous pO_2_. Fourthly, every 10 min during perfusion, a wash cycle enhances the surface oxygenation efficiency.

### 3.4. Duration of Active Oxygenation during HMP

The duration of active oxygenation during HMP to obtain optimal mitochondrial protection is not yet determined. Based on unpublished preclinical data, a new oxygenation concept during HMP is currently under investigation in a clinical trial (ClinicalTrials.gov identifier: NCT05430620) [69]. Kidneys are randomized in two treatment arms: intermittent surface oxygenation (active oxygenation is interrupted during organ transport) versus continuous surface oxygenation (control group). The purpose is to assess the effect of oxygen interruption during organ transport of HMP-perfused kidneys on metabolic status at the end of the preservation period and on initial graft function. Indeed, the elimination of an O_2_ source and membrane oxygenator during organ transport might result in a decrease in the economic and ecological costs of HMPO_2_.

### 3.5. HMP as a Tool for Kidney Assessment Prior to Transplantation

Different markers in the preservation fluid have been assessed and evaluated to predict organ quality and post-KT outcome [44,71,72,73]. In the Eurotransplant machine perfusion trial, glutathione-S-transferase, N-acetyl-β-D-glucosaminidase, and heart-fatty acid binding protein predicted DGF in an independent manner. Nevertheless, this was not the case for PNF and graft survival [74]. According to a systematic review, glutathione-S was the most reliable biomarker for predicting DGF [75]. However, its predictive potential was rather moderate [75]. The main disadvantages of these markers are their limited predictive value of post-KT outcomes and the logistic issues for implementation in daily practice.

In liver transplantation, the extent of FMN release measured by simple fluorescence of the perfusate was correlated with graft outcome [76]. A recently published porcine perfusion model demonstrated the feasibility of FMN measurement in kidneys during oxygenated HMP. FMN quantification was correlated with pre-existing kidney graft injury (see also Figure 1 for a detailed working mechanism) [77]. In the 60 min warm ischemia time (WIT) group, FMN release was significantly higher compared to the 30 min WIT and the control group and also correlated also with damage-associated molecular pattern (DAMP) signaling, such as 8-OHdG and HMGB1. ATP replenishment was demonstrated to be the best in control kidneys, followed by 30 min WIT kidneys and finally 60 min WIT kidneys. Based on these data, FMN could be potentially used as a real-time surrogate marker to assess metabolic status and predict IRI. Therefore, FMN could be used as an objective assessment tool to accept high-risk grafts for KT.

### 3.6. Clinical Evidence for HMPO_2_: End-Ischemic versus Continuous Perfusion

Currently, six clinical trials studied the effect of active oxygenation during HMP with a variety of O_2_ administration strategies, as detailed in Table 1 [63,65,66,78,79,80]. The strongest evidence for active oxygenation during HMP is derived from the multicenter randomized controlled trial (RCT) including 50 years or older DCD Maastricht category III donors. Patients were randomized to receive either a kidney following continuous oxygenated HMP (HMPO_2_) or non-oxygenated HMP. This double-blinded study contained 106 kidney pairs perfused during the entire preservation period. The primary outcome of the 12-month estimated glomerular filtration rate (eGFR) was similar between both study groups, but the graft failure rate at one year after transplantation was significantly lower in the oxygenated group as compared to the non-oxygenated HMP group (3% versus 10%, *p* = 0.028) [65]. These positive findings could not be confirmed in two matched-case studies. Different studies assessing the effects of end-ischemic HMPO_2_ in ECD DBD grafts could not demonstrate an improvement in DGF and PNF rate nor early graft function and biopsy-proven acute rejection rate as compared to SCS alone [63,66,78,79,80].

**Table 1:** Overview of clinical studies using end-ischemic or continuous oxygenated hypothermic machine perfusion for kidney preservation.

	Meister FA et al., 2019 [78]	Ravaioli M et al., 2020 [63]	Jochmans I et al., 2020 [65]	Husen P et al., 2021 [66]	Houtzager J et al., 2021 [79]	Pravisani R et al., 2022 [80]
Donor type	ECD DBD	ECD DBD	DCD > 50 y	ECD DBD	DBD DCD	NA
Perfusion device	Kidney Assist Transporter (Organ Assist)	Unique device developed by Medica S.P.A and Centro Iperbarico S.R.L.	Kidney Assist Transporter (Organ Assist)	Kidney Assist Transporter (Organ Assist)	Oxygenated Airdrive HMP system	Waves Machine
Type of study	Matched-case analysis	Matched-case analysis	RCT (COPE-COMPARE trial)	RCT (COPE-POMP trial)	Phase I	Retrospective study
Study groups (n = included patients)	1. SCS + HMPO_2_ (n = 15) 2. SCS (n = 30) (historical cohort group)	1. SCS + HMPO_2_ (n = 10) 2. SCS (n = 30) (historical cohort group)	1. HMPO_2_ (n = 106) 2. HMP (n = 106)	1. SCS + HMPO_2_ (n = 127) 2. SCS (n = 135)	1. SCS(n = 4)/ HMP (n = 1) + HMPO_2_	1. SCS + HMPO_2_ (21%0_2_) (n = 51) 2. SCS + HMP (n = 52)
Donor age, years						
Study group 1	66.0 (±12) *	71.5 (60–78)	58.0 (54–63)	64.0 (50–82)	44.2 (19–64)	62.0 (44–73)
Study group 2	66.0 (±8) *	69.5 (59–79)	58.0 (54–63)	65.0 (51–84)	-	60.0 (48–70)
Donor WIT, min						
Study group 1	-	-	28.8 (22–36)	34.0 (17–92)	NA	50 (37–59)
Study group 2	-	-	28.8 (22–36)	32.0 (10–80)	-	44 (35–52)
Duration on MP, hour						
Study group 1	2.5 ± 1.5 *	Mean 3.3 (1–6 h)	11.0 (8.7–13.7)	4.7 (0.8–17.1)	8.5 (3–15)	20.3 (18.1–22.7)
Study group 2	-	-	10.3 (8.9–14.0)	-	-	20.3 (18.1–22.3)
*p*-value	NA	NA	0.410	NA	-	0.678
CIT, hour						
Study group 1	10.8 ± 3.8 *	14.5 (10.8–22)	6.85 (4.5–9.1)	13.2 (5.1–28.7)	20.2 (11–29.5)	29 (26.6–31)
Study group 2	11.2 ± 3.6 *	14 (8–21)	7.40 (4.8–9.9)	12.9 (4–29.2)	-	29.8 (27.6–31.5)
*p*-value	0.563	0.896	0.210	NA	-	0.438
DGF rate, n (%)						
Study group 1	8 (53%)	2 (20%)	38 (36%)	30 (23.6%)	3 (60%)	11 (21.5%)
Study group 2	10 (33%)	12 (40%)	38 (36%)	38 (28.1%)	-	13 (25%)
*p*-value	0.197	0.607	0.990	0.400	-	0.648
PNF rate, n (%)						
Study group 1	1 (7%)	0 (0%)	3 (3%)	8 (6.3%)	1 (20%)	NA
Study group 2	0 (0%)	1 (3.3%)	5 (5%)	8 (5.9%)	-	NA
*p*-value	0.333	0.948	0.480	0.900	-	NA
eGFR (mL/Min/1.73 m^2^)	(At 6 mo)		(At 1 year)	(At 1 year)		(At 1 year, serum creatinine level)
Study group 1	32 ± 14 *	NA	50.5 ± 19.3 *	39.9 ± 14.4 *	NA	1.27 mg/dL
Study group 2	38 ± 17 *	NA	46.7 ± 17.1 *	41.2 ± 17.1 *	-	1.40 mg/dL
*p*-value	0.276	NA	0.120	0.530	-	0.319
Graft survival, %	(At 6 months)	(At 1 year)	(At 1 year)	(At 1 year)		(At 1 year)
Study group 1	93%	100%	10%	92.1%	NA	96.1%
Study group 2	100%	93.3%	90%	93.3%	-	100%
*p*-value	0.333	0.894	0.028	0.630	-	0.495
Postoperative complications, % (Clavien-Dindo grade 3 or more)						
Study group 1	NA	NA	11%	NA	NA	15.7%
Study group 2	NA	NA	13%	NA	-	11.5%
*p*-value	NA	NA	0.032	NA	-	0.775
BPAR						
Study group 1	NA	NA	15 (14%)	23 (18.1%)	NA	6 (11.7%)
Study group 2	NA	NA	27 (26%)	18 (13.3%)	-	4 (7.7%)
*p*-value	NA	NA	0.040	0.290	-	0.741

Abbreviations: BPAR, biopsy-proven acute rejection; COPE: Consortium for Organ Preservation; DBD, donation after brain death; DCD, donation after circulatory death; DGF, delayed graft function; ECD, extended criteria donor; eGFR, estimated glomerular filtration rate; HMP, hypothermic machine perfusion; HMPO_2_, oxygenated hypothermic machine perfusion; MP, machine perfusion; NA, not available; PNF, primary nonfunction; RCT, randomized clinical trial; SCS, static cold storage. Data are presented as median (range), except *. Table and table legend are an updated version of Table 2 of T. Darius et al. [35].

## 4. Normothermic Machine Perfusion 

### 4.1. Establishment of Normothermic Machine Perfusion of the Kidney

In 1934, Charles Lindbergh and Alexis Carrel developed the first machine to preserve animal organs, thereby introducing ex vivo normothermic machine perfusion (EVNP). However, EVNP disappeared for decades because of the simplicity, low cost, and excellent results of SCS alone in times when donor shortage was rare [81]. During the last decade, there has been renewed interest in perfusing organs for transplantation with warm solutions. NMP offers a strategy to perfuse grafts under near-physiological conditions, increasing metabolic activity and restoring the cellular energy profile [82,83,84]. NMP of the kidney uses oxygenated red blood cells or acellular solutions containing a variety of supplements and additives to restore oxidative phosphorylation and protect the vasculature against the inflammatory and vasoconstrictive environment induced during IRI. Early animal studies of canine autotransplantation and porcine kidney perfusion demonstrated the feasibility and potential reconditioning benefits of machine perfusion at approximate body temperature (37 °C) [85,86,87,88,89]. The bridge to clinical practice was established in 2011, with the first case of NMP in human KT undertaken in Leicester by Hosgood and Nicholson, ushering in a new era of kidney preservation in organ transplantation [90]. Clinical use of NMP has since been established in more centers around the world, although implementation in standard kidney preservation practice has yet to be achieved.

### 4.2. NMP as an Alternative to Cold Preservation

Normothermic machine perfusion has many potential benefits for organ preservation compared to its hypothermic counterpart. Restoration of normoxic and normothermic conditions promotes cellular metabolism and repair processes and oxidative phosphorylation to replenish ATP levels, mimicking a near-physiological environment [82,83,84,85,91]. The superiority of NMP compared to HMP, however, has yet to be established, although a clinical trial is currently underway in the Netherlands to compare graft outcome of extended criteria DBD and DCD donor kidneys using both strategies [92]. During ischemic hypothermia, cellular ATP depletion occurs, and basic cell function is maintained by glycolysis [16,53,93]. An accumulation of ROS results in increasing oxidative damage as cold ischemia continues, contributing to mitochondrial dysfunction and tubular injury [16,94]. While oxygenated HMP has been suggested as a strategy to prevent this injury, hyperoxia is likely associated with an increase in ROS production, and so careful consideration is required. In contrast, oxygenated normothermia restores mitochondrial activity and oxidative phosphorylation, alongside re-establishing the activity of ROS scavengers. Directed delivery of potent antioxidants within the perfusate can also be achieved to ameliorate oxidative stress. NMP is an effective means of preserving mitochondrial function and ATP replenishment ex situ. Current logistical challenges have limited the implementation of NMP for kidney preservation compared to HMP [95]. The only commercially available NMP device at this moment for the kidney is the Kidney Assist™, produced by Organ Assist (Organ Assist BV, Groningen, The Netherlands), although further devices are in development. Further limitations center around the lack of universally adopted reagents and protocols, as is expected with a rapidly advancing new technology, and presents difficulties in comparing results across studies from different centers.

Indeed, a focus of present NMP studies of kidney preservation is the optimization of a perfusate for the restoration of near-physiological conditions. Due to the increased metabolic demand for oxygen and nutrients, alongside vascular challenges in response to inflammation, microbial contamination, and electrolyte imbalance, there is currently no consensus for a standardized perfusate composition. Almost all clinical studies to date have used a leukocyte-depleted red blood cell-based solution with a variety of added nutrients, supplements, additives, and antibiotics [96]. Some studies have used a colloid-based solution to buffer against cellular edema, although this has not been applied in all centers. A systematic review by Fard et al. highlighted the huge heterogeneity across all the experimental and clinical studies investigating NMP of the kidney that precluded meta-analysis [96]. Drawing robust conclusions about ideal perfusate composition and NMP variables is, therefore, a difficult undertaking, particularly when considering the different NMP systems, length of perfusion, and outcome measures evaluated in different centers. Studies of porcine kidney NMP have investigated perfusate composition in more depth, comparing a variety of existing perfusate solutions and the possibility of a red cell-free system [97,98]. Small animal group sizes and lack of post-NMP transplantation and follow-up data limit the clinical translation of these studies to a universal perfusate composition.

### 4.3. NMP as a Tool for Kidney Assessment Prior to Transplantation

Ex situ assessment of a kidney during NMP has clear advantages compared to the assessment of preservation solution-flushed cold storage organs. For most standard criteria transplanted kidneys whereby a modest cold ischemia time is achievable, pretransplant viability assessment is not necessary. However, there is evidence that higher-risk organs (DCD and DBD ECD kidneys) are more likely to be poorly flushed with preservation solution in situ and thus are less likely to deemed suitable for transplantation [99]. A period of NMP would seem invaluable for such organs and has been shown to be able to restore function to marginal organs, DCD kidneys and a case of poor perfusion of a single kidney after normothermic regional perfusion, all of which were previously deemed unsuitable for transplantation and have subsequently shown suitable function in the recipients [99,100,101]. Hosgood et al. developed the Quality Assessment Score (QAS), an assessment tool based on NMP parameters, macroscopic appearance, and urine output during perfusion of over 40 human kidneys declined for KT [102]. The QAS was then applied in a clinical series of 10 kidneys initially rejected for transplantation, where 5 were then transplanted successfully, with only one instance of DGF reported. So, ex situ assessment of kidneys during NMP has been shown to direct clinical decision making with suitable outcomes and could be implemented as a routine procedure for marginal kidneys that may otherwise be declined for transplantation.

Perhaps unsurprisingly given the high metabolic requirement of a kidney in normothermic conditions, a variety of additional markers have been reported to assess organ quality. These include viability via measures of oxygen consumption [103,104,105], inulin clearance [106], gene expression changes in tissue biopsies [82], and the production of biomarkers [100,107,108,109,110]. The use of biofluids produced during NMP as a source of non-invasive soluble biomarkers to predict graft outcome has been largely investigated in recent years. For example, FMN, a marker of mitochondrial damage, has been measured in the NMP perfusate of a clinical series of human kidneys perfused prior to transplantation, showing a significant increase in allografts that went on to develop DGF and PNF [108]. Characterization of extracellular vesicles extracted from the perfusate of human kidneys undergoing NMP could be a useful non-invasive tool to further assess the quality of kidneys prior to transplantation [110,111].

### 4.4. Clinical Evidence for NMP: End-Ischemic versus Continuous Perfusion

NMP today is mostly used as an end-ischemic preservation strategy following a preceding SCS or HMP period. Continuous NMP after classical kidney procurement has not yet been realized until now. However, an even more optimal strategy is to avoid ischemia-reperfusion injury by not stopping the blood supply before organ procurement in the donor. This technique is called “ischemia-free organ transplantation” and was published in 2019 for the first time in KT by He X et al. [112]. This strategy looks promising. However, it has not yet been repeated because of major logistic limitations.

Several clinical series have been published using EVNP since the publication in 2011 of the first human KT after end-ischemic NMP and summarized in Table 2 [90]. In 2013, Nicholson ML and Hosgood SA reported the first series of kidney transplants performed after a period of EVNP [113] (Table 2). As compared to SCS alone, EVNP kidneys demonstrated a significant reduction in DGF (5.6 M vs. 36.2%, *p* = 0.014) [113]. Chandak P et al. failed to demonstrate a difference in clinical outcome comparing seven end-ischemic EVNP kidneys with seven contralateral kidneys (from the same DCD donor) preserved by classical SCS alone [95]. In 2021, the Rotterdam group transplanted 11 kidneys after 120 min of EVNP. However, an additional 1 hour of EVNP, compared to both previous clinical series of Nicholson ML [113] and Chandak P [95], demonstrated no difference in PNF (0% vs. 8%, *p* = 0.347) and DGF (36% vs. 53%, *p* = 0.320) as compared to classic SCS or HMP [114]. These transplants were performed in the context of the Eurotransplant Senior Program, in which kidneys from a donor aged 65 years or above are allocated to recipients aged 65 years or more. The last published series until now was published by Mazilescu L et al. in 2022, and EVNP kidneys with even longer machine perfusion time were compared to matched anoxic HMP ones [115]. No difference in initial clinical outcome was observed between both groups.

**Table 2:** Overview of clinical studies using end-ischemic normothermic machine perfusion for kidneys.

	Nicholson M. et al., 2013 [113]	Chandhak P. et al., 2019 [95]	Rijkse E. et al., 2021 [114]	Mazilescu L. et al., 2022 [115]
Donor type	ECD (1 DCD + 17 DBD)	DCD	ECD (7 DCD + 4 DBD)	7 DCD + 6 DBD
Perfusion device	Designed on pediatric cardiopulmonary bypass technology (Medtronic)	Designed on pediatric cardiopulmonary bypass technology (Medtronic)	Kidney Assist	Designed on pediatric cardiopulmonary bypass technology (Medtronic)
Type of study	Cohort study	Clinical series	Clinical series	Clinical series
Study groups (n = included patients)	SCS + EVNP (n = 18) SCS (n = 47)	SCS + EVNP (n = 7) SCS (n = 7) (contralateral kidney)	SCS/HMP (3/8) + EVNP (n = 11) SCS/HMP (9/44) (n = 53)	HMP + EVNP (n = 13) HMP (n = 26) (matched)
Donor age, years				
Study group 1	61 (60–62)	48 (29–61)	71 (66–72)	61 (52–66)
Study group 2	62 (56–68)	57 (31–61)	69 (68–73)	60 (51–64)
Donor WIT, min				
Study group 1	26 (20–32)	NA	13 (0–17) + 18 (17–21)	29 (26–35)
Study group 2	31 (27–35)	NA	13 (2–15) + 22 (17.5–28.5)	32 (30–36)
Duration on EVNP, min (Temp, °C—02 conc, % —MP pressure, mmHg)	63 (47–79)	60	120	171
Study group 1	(36 °C—100% O_2_—52/70 mmHg)	(36 °C—100% O_2_—75 mmHg)	(37 °C—100% O_2_—60 mmHg)	(37 °C—100% O_2_—65 mmHg)
CIT, min				
Study group 1	774	838	593	537
Study group 2	738	608	600	616
*p*-value	0.614	0.010	0.893	0.450
DGF rate, n (%)				
Study group 1	1 (5.6%)	1 (14%)	4 (36%)	4 (30.8%)
Study group 2	17 (36.2%)	3 (43%)	28 (53%)	10 (38.5%)
*p*-value	0.014	0.560	0.320	0.733
PNF rate, n (%)				
Study group 1	0 (0%)	0 (0%)	0 (0%)	0 (0%)
Study group 2	1 (2%)	1 (14%)	4 (8%)	0 (0%)
*p*-value	1.000	1.000	0.347	1.000
eGFR (ml/min/1.73 m^2^)		At 1 year	At 1 year	At 1 year
Study group 1	NA	59	>30: 6 (55%)	75.3
Study group 2	NA	53	>30: 31 (58%)	76.5
*p*-value	NA	0.640	0.809	0.595
Graft survival, %	At 1 year	At 1 year	At 1 year	At 1 year
Study group 1	100%	100%	91%	100%
Study group 2	96%	86%	91%	96%
*p*-value	1.000	0.310	0.537	0.480
Postoperative complications, % (Clavien-Dindo grade 3 or more)				
Study group 1	NA	NA	NA	23.1
Study group 2	NA	NA	NA	11.5
*p*-value	NA	NA	NA	0.380
BPAR				
Study group 1	5 (27.7%)	NA	4 (36%)	NA
Study group 2	11 (23.4%)	NA	14 (26%)	NA
*p*-value	0.753	NA	0.504	NA

Abbreviations: BPAR, biopsy-proven acute rejection; DBD, donation after brain death; DCD, donation after circulatory death; DGF, delayed graft function; ECD, extended criteria donor; eGFR, estimated glomerular filtration rate; EVNP, ex vivo normothermic perfusion; HMP, hypothermic machine perfusion; MP, machine perfusion; NA, not available; PNF, primary nonfunction; SCS, static cold storage.

## 5. Future Perspectives of HMPO_2_ and NMP in Clinical Practice

Active oxygenation during HMP has been demonstrated to be feasible, safe, and easy to implement in clinical practice, independent of the O_2_ administration technique. However, clinical evidence for active oxygenation during HMP to improve preservation and clinical outcome is still limited. HMPO_2_ is most beneficial in DCD kidneys when applied from the moment of kidney procurement until transplantation and not as an end-ischemic strategy [65,66]. Larger clinical series, RCTs, or large registries need to confirm the impact. Real-time FMN detection by spectrometry may be a promising surrogate marker for mitochondrial damage assessment to further increase the utilization rate of high-risk kidneys.

Perhaps the most promising clinical application of NMP is the therapeutic potential of direct targeting of the kidney ex situ without the risk of systemic or off-target effects in a potential recipient. In contrast to HMP, kidneys perfused in normothermic conditions retain a near-physiological metabolic profile, allowing rapid uptake and processing of drugs and metabolites, which is substantially reduced in hypothermia. Recent advances have been made in the field of regenerative medicine, with the application of stem cell therapies to recondition and repair kidneys, potentially improving the quality and therefore enabling further utilization of marginal or extended criteria organs [116,117,118,119,120,121]. Directed renal therapies have already been shown to ameliorate microvascular obstructions, reduce inflammation, and improve AKI in animal and human kidney models [82,122,123]. Precise targeting of nanoparticles to cell-specific compartments may further personalize drug delivery to organs ex situ during NMP [124]. NMP has also been used to apply antisense oligonucleotide therapy as a way to target the destruction of key pathogenic microRNAs in the renal tubular epithelium, where HMP conditions, in contrast, resulted in poor cellular uptake of the therapy [125]. Pre-transplantation removal of blood group antigens during NMP may also be able to remove the ABO barrier in organ allocation [126].

In a clinical context, current NMP strategies have been focused on optimizing the preservation of organs and providing safety and feasibility data [92,95,113,114,115,127,128]. However, results of the multicenter U.K. trial [129], as well as the ongoing clinical trial in the Netherlands [90] and Germany (ClinicalTrials.gov identifier: NCT05031052), are still pending.

One of the crucial limitations to implementing new machine preservation strategies is the cost and the lack of cost-effectiveness analysis. Few studies demonstrate the cost-effectiveness of HMP as compared to SCS alone [55]. NMP is more complex and requires more logistics compared to HMPO_2,_ but currently, cost-effectiveness analyses for NMP are missing.

## 6. Conclusions

HMPO_2_ and NMP are novel and promising preservation strategies. Based on current clinical evidence and compared to static cold storage, HMPO_2_ improves preservation of the graft in the cold. During HMPO_2,_ FMN might be a potential real-time biomarker correlating with the underling graft injury as well as predicting post-transplant outcomes. One of the main advantages of NMP is the potential to use this perfusion approach as a platform to assess novel therapies for kidney modulation and as an ex vivo assessment tool for high-risk kidneys to decrease the discard rate of high-risk kidneys, maybe in combination with other perfusion approaches, e.g., HMPO_2,_ to change the mitochondrial state followed by long-term NMP for graft assessment. Therefore, HMPO_2_ and NMP must be seen as two complementary new techniques with the potential to change the future of organ preservation and transplantation.

## Data Availability

Not applicable.

## Abbreviations

ADP	adenosine diphosphate
AMP	adenosine monophosphate
ATP	adenosine triphosphate
CI	(mitochondrial) complex I
CAC	citric acid cycle
CIT	cold ischemia time
DAMP	damage-associated molecular pattern
DBD	donation after brain death
DCD	donation after circulatory death
DGF	delayed graft function
ECD	expanded criteria donor
EVNP	ex vivo normothermic perfusion
FET	forward electron transport
FMN	flavin mononucleotide
FMNH2	flavin mononucleotide release
eGFR	estimated glomerular filtration rate
HMGB1	high-mobility group box 1
HMP	hypothermic machine perfusion
HMPO2 IRI	Oyxgenated hypothermic machine perfusion ischemia-reperfusion injury
KT	kidney transplantation
NAD^+^	oxidized nicotinamide adenine dinucleotide
NADH	reduced nicotinamide adenine dinucleotide
NMP	normothermic machine perfusion
PNF	primary nonfunction
pO_2_	partial pressure of oxygen
QAS	quality assessment score
QH2	Ubiquinol
ROS	reactive oxygen species
RCT	randomized clinical trial
RET	reversed electron transport
SCS	static cold storage
WIT	warm ischemia time
